# Predictors of Mortality Among Post-COVID-19 Discharged Patients in Northern India: A Case-Control Study

**DOI:** 10.7759/cureus.36883

**Published:** 2023-03-29

**Authors:** Arjun Kumar, Basavaraj Jatteppanvar, Prasan K Panda, Pathik Dhangar, Yogesh A Bahurupi

**Affiliations:** 1 Internal Medicine, All India Institute of Medical Sciences, Rishikesh, IND; 2 Community and Family Medicine, All India Institute of Medical Sciences, Rishikesh, IND

**Keywords:** icu admission, acute kidney injury, risk factors, post-discharge, covid mortality

## Abstract

Introduction

The post-discharge all-cause mortality of COVID-19 disease is known, but predictors for the same have not been studied as much. The objective of this study was to develop an understanding of predictors of mortality to guide in prioritizing patient care and preventive approaches.

Methods

This current research is a single-center unmatched case-control study conducted at a tertiary care center in northern India, between April and September 2022. The data were extracted retrospectively from the hospital's electronic medical records of patients with the assistance of trained physicians using a standardized data extraction sheet.

Results

A total of 184 patients were enrolled and were segregated into two groups, cases and control, with 92 in each. The mean age of patients was 49.3 ± 17.53 years. The mortality group had a higher mean age (53.24 ± 18.53 yrs) as compared to the control group (45.37 ± 15.58 yrs, p=0.002). Bivariate analysis revealed a significant difference in the two groups with respect to O_2_ saturation at the time of admission (case - 91.12 ± 12.49 %, control - 95.46 ± 5.01 %, p=0.003); maximum O_2_ flow rate (L/min) (case - 11.01 ± 22.2, control - 6.41 ± 13.31, p=0.04); ICU need (p=0.005), cancer (p=0.001), O_2_ requirement at discharge (p=0.001) and acute kidney injury (AKI; p=0.007). On multiple regression analysis, cancer (adjusted odds ratio (aOR) - 2.469; 95% CI 1.183-5.150, p=0.016), ICU admission (aOR - 2.446; 95% CI 1.212-4.938, p=0.013), oxygen at discharge (aOR - 2.340; 95% CI 0.971-5.640, p=0.0586) and AKI (aOR - 5.6; 95% CI 2.351- 13.370, p=0.00) only found to be significant.

Conclusion

Among the patients released from the hospital post-COVID-19 treatment, the following aspects oxygen requirement (2.3 times), malignancy (2.4 times), ICU admission (2.4 times), and AKI (5.6 times) are risk factors of mortality. The presence of these variables would warrant a close follow-up for these patients in order to decrease post-COVID mortality.

## Introduction

The global pandemic of severe acute respiratory syndrome coronavirus (SARS-CoV-2) or COVID-19 has caused more than six million deaths, and India has been one of the most severely affected countries, with 530,808 deaths reported by March 23, 2023 [1, 2]. The rapid increase in the rate of vaccination across the globe has contributed to a decline in the incidence; however, substantial mortality still exists [3]. The emergence of new strains of the virus increases the likelihood of repeat surges of cases and deaths due to the disease.

The predictors of morbidity and mortality have been well studied for acute COVID-19 disease in hospitalized patients, but similar data regarding post-COVID-19 illness remains to be lacking.

Outpatient post-COVID-19 data suggests the persistence of multiple symptoms in this vulnerable population and the development of new cardiovascular and pulmonary complications [4]. All-cause 30-day mortality and 30-day readmission rate of post-COVID-19 patients discharged on home-based oxygen therapy were reported to be 1.3% and 7.5%, respectively, in a retrospective cohort study [5]. These figures may differ significantly in an Indian setting, as has been described in the literature for acute COVID-19 illness [6]. Ethnicity, gender, comorbidities, and severity of symptoms, amongst others, determined the mortality and morbidity of an acute COVID-19 illness [7].

Such understanding of the predictors helps in prioritizing patient care and preventive approaches. However, similar data regarding post-COVID-19 mortality has been scarce. There is limited data from India regarding the prevalence and predictors of post-COVID-19 mortality. This single-center hospital-based study was done with the objective of identifying the predictors of mortality among symptomatic COVID-19 patients discharged from the hospital setting.

## Materials and methods

This was a single-center hospital-based unmatched case-control study conducted at a tertiary care center, All India Institute Medical Sciences (AIIMS), in Rishikesh, India. The patients diagnosed positive through COVID-19 real-time reverse transcriptase polymerase chain reaction (RT-PCR) who were discharged from the hospital during the study duration and died within a three-month period after discharge were considered as cases. On the other hand, patients who were alive for the three-month post-discharge period were considered controls. Records of all the COVID-19 patients discharged from the hospital were entered in the form of a data extraction sheet, and all the patients were followed up telephonically at three months. The records were updated as alive or death responses along with the date of death in case of mortality. All the patients discharged during the duration of the study were included irrespective of the discharge status being leave against medical advice, discharge on patient request, or normal discharge. The study was conducted over 16 months from April 2021 to September 2022. All patients affected by COVID-19, who were over the age of 18, were admitted to the hospital, and died during follow-up were considered for the study. The controls were unmatched and were selected randomly from the hospital records in a ratio of 1:1. All the information related to the controls and cases was extracted retrospectively from the medical records of patients.

Patient-related information was extracted by trained physicians using a standardized data extraction sheet. The data extracted included age, sex, smoking, alcohol consumption, and comorbidities like hypertension, diabetes, heart disease, mucormycosis, cancer, chronic kidney disease, and heart disease. Vitals at the time of presentation, such as heart rate, respiratory rate, and blood pressure, were extracted. Data regarding symptom duration before admission, duration of hospital stay, saturation at admission, maximum O2 requirement at admission, type of respiratory support (nasal prongs, face mask, non-rebreathing mask, high flow nasal cannula, non-invasive ventilation, invasive ventilation), duration of oxygen requirement in hospital and need for oxygen at discharge was collected. Imputation was not performed for missing data. All the study variables were represented either as means or proportions and compared between cases and controls using a Chi-square test or unpaired t-test, wherever appropriate. Univariable and multivariable logistic regression was done to assess the association between various predictor variables and deaths due to COVID-19. A p<0.05 was considered significant. Ethical approval for the analysis was obtained from the Ethics Committee of the All-India Institute of Medical Sciences, Rishikesh, and all participating institutes (CTRI/2020/08/027169). This article was previously posted to the medRxiv preprint server on March 08, 2023.

## Results

A total of 184 patients were included in the study; 92 were cases, and 92 were controls. The mean age of patients was 49.3 ± 17.53 years. A relatively high proportion of 65.46% were males, and 34.54% were females. Cough was the most common symptom at the time of admission (53.1%), followed by dyspnea, which was present in 31.44% of cases. The mean duration of symptom onset before presentation to the hospital was 4.41 ± 3.85 days, and the mean hospital stay was 12.12 ± 10.61 days. At least 34.53 % of patients required oxygen need at admission, while 40.20% required oxygen anytime during the hospital stay. A smaller proportion of 24.74% of patients required ICU care, with 4.63% of all requiring vasopressor support. Around 4.63% received invasive ventilation, while 8.24% received non-invasive ventilation, and 10.81% of all patients required oxygen at discharge (Table 1).

**Table 1:** Demographic and clinical characteristics of patients and controls

Characteristics	Total (n=194)	Cases (n=92)	Controls (n=92)	p-value
Age (years)	49.3 ± 17.53	53.24 ± 18.53	45.37 ± 15.58	0.002
Sex (M/F)	127/57	64/28	63/29	0.5
Symptom duration before admission (days)	4.41 ± 3.85	4.46 ± 4.43	4.35 ± 3.25	0.848
Duration of hospital stay (days)	12.12 ± 10.61	11.59 ± 11.10	12.65 ± 10.12	0.498
Saturation at admission (%)	93.27 ± 9.76	91.12 ± 12.49	95.46 ± 5.01	0.003
Max flow (L/min)	8.55 ± 18.15	11.01 ± 22.2	6.41 ± 13.41	0.04
Duration of O_2 _in hospital (days)	4.88 ± 6.93	4.65 ± 6.84	5.08 ± 7.03	0.715
Duration of ICU stay (days)	4.04 ± 8.13	4.92 ± 9.16	3.31 ± 7.14	0.249
O_2_ flow at discharge (L/min)		1.50 ± 4.12	0.03 ± 0.183	0.007
O_2_ need at admission	67	33	34	0.5
O_2_ needed during admission	78	38	40	0.466
Vasopressor need	9	7	2	0.077
ICU need	48	31	6	0.005
Dyspnoea	61	44	17	0.001
Hypertension	49	24	25	0.536
Diabetes	43	22	21	0.45
Mucor	8	5	3	0.33
Cancer	26	20	6	0.001
Heart disease	24	12	12	0.528
Chronic kidney disease	21	14	7	0.059
O_2_ need at discharge	21	19	2	0.001
Acute kidney injury	13	11	2	0.007

On bivariate analysis, as compared to controls, the cases were more likely to be older patients (53.24 years vs. 45.37 years, p=0.002), with comorbidities (cancer - 21.73% vs. 6.5%, p=0.001; chronic kidney disease - 15.21% vs. 7.60%, p=0.05). Cases were more likely to experience dyspnea (47.82% vs. 18.47%, p=0.001) and lower saturation (91.12% ± 12.49 vs. 95.46 ± 5.01%, p=0.003) at the time of admission as compared to controls. Over the course of hospital stay, the cases were more likely to have higher oxygen flow rates (11.01 ± 22.2 L/min vs. 6.41 ± 13.41 L/min, p=0.04), high demand for ICU admission (33.69% vs. 6.50%, p=0.005), development of AKI (11.95% vs. 2.17%, p=0.007) and higher incidence of vasopressor need (7.60% vs. 2.17%, p=0.07) as compared to controls. Cases presented the need for a higher probability of invasive ventilation (7.60% vs. 2.17%, p=0.05) and non-invasive ventilation (10.86% vs. 6.52%, p=0.04). At the time of discharge, the cases were relatively more likely to require oxygen at discharge (20.60% vs. 2.17%, p=0.007) than controls (Table 1).

Upon multivariable analysis, ICU need (adjusted odds ratio (aOR) 2.44, 95% CI 1.212-4.938), O2 requirement at discharge (aOR 2.34, 95% CI 0.971-5.640), cancer (aOR 2.46, 95% CI 1.18-5.15) and AKI during hospital stay (aOR 5.60m 95% CI 2.35-13.37) were observed to be associated with post-COVID-19 mortality. Multicollinearity was tested with linear regression and creating a correlation matrix. None of the variables showed a high correlation coefficient. After adjusting for all variables, sex, vasopressor need, and comorbidities, including mucormycosis, hypertension, and diabetes, were not associated with increased post-COVID-19 mortality (Table 2).

**Table 2:** Association between demographic, clinical characteristics, and mortality among COVID-19 patients discharged from the hospital Using multiple regression analysis to estimate adjusted odds ratio (aOR) and 95% confidence interval (CI)

Variable	aOR	95% CI	p-value
Age	1.03	1.00 – 1.06	0.01
Sex	1.29	0.72 – 2.33	0.38
ICU need	2.44	1.21 – 4.93	0.01
Vasopressor need	0.94	0.25 – 3.41	0.92
Need for O_2_ at discharge	2.34	0.97 – 5.64	0.05
Acute kidney injury	5.60	2.35 – 13.3	0.01
Malignancy	2.46	1.18 – 5.15	0.01
Diabetes	1.17	0.56 – 2.41	0.66
Mucor	1.26	0.27 – 5.95	0.76
Hypertension	0.79	0.40 – 1.57	0.51

## Discussion

In this hospital-based control study on predictors of mortality in patients discharged from a hospital setting after COVID-19 illness, we found that older age, oxygen saturation at admission, maximum oxygen requirement during the hospital stay, flow rate at discharge, ICU admission need during the hospital stay, cancer, chronic kidney disease, and acute kidney injury during hospital stay; indicated increased mortality post-discharge. This study adds up to the current data regarding the prognosis and follow-up of post-COVID patients.

In this study, older age has been noted as an independent factor for increased mortality among COVID patients discharged from the hospital. We found that with increasing age (aOR 1.033, 95% CI 1.007-1.061) was associated with increased odds of mortality after being discharged from COVID-19 illness. A long-term mortality study in Estonia also noted a similar finding with increased one-year mortality in patients older than 60 years [8]. In-hospital mortality of COVID-19 illness also revealed similar findings in a meta-analysis of 611,583 patients (OR 3.13, 95% CI 2.61-3.76) [9]. A similar association was found in other meta-analyses and studies conducted in other countries [10]. The association between sex and mortality has not been well defined in studies in both hospitalized COVID-19 patients and discharged patients, and the results have been conflicting. In the present study, we also did not find any correlation between sex with mortality.

Poor clinical status, which comprised of patients having more oxygen requirement at admission and requiring higher flow rates of oxygen during the hospital stay, was associated with increased post-COVID mortality. The patients who needed ICU admission or vasopressor need at any time during the hospital stay also had increased odds of mortality (aOR 2.44, 95% CI 1.212-4.938). A study of 13,638 patients on follow-up after COVID-19 illness revealed increased 12-month mortality in patients having severe COVID-19 infection (HR 2.50; 95% CI 2.02-3.09). Similar studies done in hospitalized patients with COVID-19 also reflect similar results. Lower oxygen saturation at admission had an increased risk of death in hospitalized COVID-19 patients also [11]. Clinical status at discharge was a significant risk factor, and the patients requiring oxygen at discharge had a higher risk of post-COVID mortality (aOR 2.34, 95% CI 0.971-5.640).

Dyspnea was the only clinical symptom associated with increased risk, but it was insignificant after adjusting for other variables. In a meta-analysis of 3578 patients with COVID-19 illness, the presence of dyspnea was associated with three times increased risk of mortality [12]. Similar data on post-COVID mortality is, however, not conclusive.

Among the pre-existing comorbidities examined in the study, malignancy was found to be the independent predictor of mortality in post-COVID patients. Another similar study found increased mortality in follow-up patients due to malignancy, cardiovascular disease, and pre-morbid respiratory disease [8]. In our study, we did not find a correlation between mortality with hypertension, diabetes, heart disease, or chronic kidney disease. Several other studies done on hospitalized patients of COVID-19 illness have found correlations between mortality with malignancy and heart disease, while some have not [11, 13].

In a hospital, complications can contribute to mortality in COVID-19 patients. In this study, we found AKI to be associated with increased post-COVID mortality (aOR 5.60, 95% CI 2.35-13.37). This aspect has been analyzed in studies on in-hospital COVID mortality, and a meta-analysis of 44 studies found that acute respiratory distress syndrome, AKI, and acute cardiac injury increased the risk [14].

This is one of the first studies from India to assess the predictors of mortality among COVID-19 patients discharged from a hospital setting. It highlights the significance of follow-up in COVID-19 patients as death can be a part of long COVID syndrome.

However, there are certain limitations. As is true for any retrospective study, the extraction of records lacked completeness. Drugs and their adverse effects have not been included in the study. Post-discharge socioeconomic factors are not considered in this case-control study due to the lack of physical visits, and it is, therefore, an unmatched study. The study participants were not divided into severity categories; however, other variables like oxygen saturation at admission, length of hospital stay, need for ventilation, vasopressors, or ICU admission were included.

## Conclusions

Increasing age, malignancy, ICU admission, AKI, and oxygen requirement at discharge were significant predictors of mortality among COVID-19-recovered patients post-discharge. The presence of these variables would warrant a close follow-up for these patients in order to decrease post-COVID mortality. Understanding predictors of mortality will help guide the ongoing care of the survivors and prioritize patient care and preventive approaches in the community.

## References

[1] Hu B, Guo H, Zhou P, Shi ZL (2020). Characteristics of SARS-CoV-2 and COVID-19. Nat Rev Microbiol.

[2] (2023). WHO coronavirus disease (COVID-19) dashboard with vaccination data. https://covid19.who.int.

[3] (2022). COVID-19 map - Johns Hopkins Coronavirus Resource Center. https://coronavirus.jhu.edu/map.html.

[4] Huang C, Huang L, Wang Y (2021). 6-month consequences of COVID-19 in patients discharged from hospital: a cohort study. Lancet.

[5] Banerjee J, Canamar CP, Voyageur C (2021). Mortality and Readmission Rates Among Patients With COVID-19 After Discharge From Acute Care Setting With Supplemental Oxygen. JAMA Netw Open.

[6] Jain VK, Iyengar K, Vaish A, Vaishya R (2020). Differential mortality in COVID-19 patients from India and western countries. Diabetes Metab Syndr.

[7] Shi C, Wang L, Ye J (2021). Predictors of mortality in patients with coronavirus disease 2019: a systematic review and meta-analysis. BMC Infect Dis.

[8] Uusküla A, Jürgenson T, Pisarev H (2022). Long-term mortality following SARS-CoV-2 infection: a national cohort study from Estonia. Lancet Reg Health Eur.

[9] Bonanad C, García-Blas S, Tarazona-Santabalbina F (2020). The effect of age on mortality in patients with COVID-19: a meta-analysis with 611,583 subjects. J Am Med Dir Assoc.

[10] Dorjee K, Kim H, Bonomo E, Dolma R (2020). Prevalence and predictors of death and severe disease in patients hospitalized due to COVID-19: a comprehensive systematic review and meta-analysis of 77 studies and 38,000 patients. PLoS One.

[11] Tian W, Jiang W, Yao J (2020). Predictors of mortality in hospitalized COVID-19 patients: a systematic review and meta-analysis. J Med Virol.

[12] Mudatsir M, Fajar JK, Wulandari L (2020). Predictors of COVID-19 severity: a systematic review and meta-analysis. F1000Res.

[13] Gupta N, Ish P, Kumar R (2020). Evaluation of the clinical profile, laboratory parameters and outcome of two hundred COVID-19 patients from a tertiary centre in India. Monaldi Arch Chest Dis.

[14] Potere N, Valeriani E, Candeloro M (2020). Acute complications and mortality in hospitalized patients with coronavirus disease 2019: a systematic review and meta-analysis. Crit Care.

